# Fetal and life course origins of serum lipids in mid-adulthood: results from a prospective cohort study

**DOI:** 10.1186/1471-2458-10-484

**Published:** 2010-08-16

**Authors:** Per E Gustafsson, Urban Janlert, Töres Theorell, Hugo Westerlund, Anne Hammarström

**Affiliations:** 1 Dept of Public Health and Clinical Medicine, Family Medicine, Umeå University, Umeå, Sweden; 2 Dept of Public Health and Clinical Medicine, Epidemiology and Global Health, Umeå University, Umeå, Sweden; 3 Stress Research Institute, Stockholm University, Stockholm, Sweden

## Abstract

**Background:**

During the past two decades, the hypothesis of fetal origins of adult disease has received considerable attention. However, critique has also been raised regarding the failure to take the explanatory role of accumulation of other exposures into consideration, despite the wealth of evidence that social circumstances during the life course impact on health in adulthood. The aim of the present prospective cohort study was to examine the contributions of birth weight and life course exposures (cumulative socioeconomic disadvantage and adversity) to dyslipidemia and serum lipids in mid-adulthood.

**Methods:**

A cohort (effective n = 824, 77%) was prospectively examined with respect to self-reported socioeconomic status as well as stressors (e.g., financial strain, low decision latitude, separation, death or illness of a close one, unemployment) at the ages of 16, 21, 30 and 43 years; summarized in cumulative socioeconomic disadvantage and cumulative adversity. Information on birth weight was collected from birth records. Participants were assessed for serum lipids (total cholesterol, low- and high-density lipoprotein cholesterol and triglycerides), apolipoproteins (A1 and B) and height and weight (for the calculation of body mass index, BMI) at age 43. Current health behavior (alcohol consumption, smoking and snuff use) was reported at age 43.

**Results:**

Cumulative life course exposures were related to several outcomes; mainly explained by cumulative socioeconomic disadvantage in the total sample (independently of current health behaviors but attenuated by current BMI) and also by cumulative adversity in women (partly explained by current health behavior but not by BMI). Birth weight was related only to triglycerides in women, independently of life course exposures, health behaviors and BMI. No significant association of either exposure was observed in men.

**Conclusions:**

Social circumstances during the life course seem to be of greater importance than birth weight for dyslipidemia and serum lipid levels in adulthood.

## Background

Since the early 1990 s, the research field of life course epidemiology has studied the health effects of biological and social exposures during different periods of life and the corresponding long-term processes spanning over the life course [1]. One line of research focuses on fetal origins of disease, with one hypothesis stating that suboptimal metabolic conditions during fetal life, e.g. indicated by lower birth weight, increase the risk for cardiovascular disease in adulthood [2]. Low birth weight has been shown to be associated with cardiovascular mortality [3,4] and to predict a number of related disorders, such as hypertension, obesity and insulin resistance [5-7]. The fetal origins hypothesis has, however, been criticized for overestimation of effects [8] and failure to consider the wealth of other exposures between birth and adulthood. One of the challenges of studying the fetal origins hypothesis is how to incorporate confounding and explanatory effects of accumulated stress over the life span into the analyses [9], which is a manifest limitation of past and present research on the field. The present study will address this issue by examining the importance of birth weight as well as social exposures during the life course, for dyslipidemia and serum lipid levels in mid-adulthood.

Early studies on the fetal origins of serum lipids and dyslipidemia indicated that lower birth weight was linked to an atherogenic lipid profile in adulthood [10-12]. However, subsequent studies have only provided weak support for birth weight as a determinant of adult lipid levels [13,14]. The association between birth weight and adult total cholesterol (TC) levels appear to be too small to be of clinical significance, as concluded in a literature review by Huxley et al [14]. Regarding the apolipoproteins (Apo) A1 and B, a few studies report Apo B to be elevated among those born small [10,15,16]. For low-density and high-density lipoprotein cholesterol (LDL-C and HDL-C, respectively), findings are inconsistent not only regarding the strength but also regarding the direction of the association, as pointed out in a review by Lauren et al [13]. Triglycerides might be the lipid providing the most consistent support the fetal origins hypothesis [13]. More recent studies report weak relationships between birth weight and adult lipid levels [16,17].

In conclusion, birth weight might only be weakly related to adult serum lipids. This has been interpreted as an argument for lifestyle during adulthood as a major determinant of atherogenic serum lipids [14]. Alternatively, weak associations between birth weight and lipids could be interpreted as an argument in favor of social pathways to cardiovascular risk, which may or may not operate through behavioral pathways. Indeed, there is an increasing awareness that the accumulation of social exposures over the life course, e.g. socioeconomic status [18] and social adversity such as inadequate housing conditions [19], economic hardship [20] and adverse childhood events [21], matters for cardiovascular health in adulthood. Social exposures in both childhood and adulthood have been shown to be related to adult lipid levels [22,23]. Some studies indicate that serum lipids are related to social life course exposures only in women [24], and to birth weight only in men [25]. Alas, the accumulation of social exposures over the life course has scarcely been studied in conjunction with birth weight, despite the explanatory or confounding role such factors might play [9]. In fact, only a minority of studies on birth weight and blood lipids have adjusted for current socioeconomic status [13], which is a substantial weakness of previous research. Delineating the relative importance of fetal and later exposures for lipid status in adulthood is necessary for public health interventions and policies directed at cardiovascular risk. The present cohort study seeks to contribute to this task, by combining data on birth weight, prospectively collected exposures on socioeconomic and other adverse social exposures from adolescence to adulthood, and measurement of serum lipid levels at 43 years of age. The aims are to examine the relative contributions of birth weight and accumulated social life course exposures to dyslipidemia and serum lipid levels in mid-adulthood.

## Methods

### Participants and procedures

The sample was based on the Northern Swedish Cohort, a 27-year prospective cohort study comprising all pupils in the ninth grade of the Swedish compulsory school living in Luleå in 1981, when the participants where 16 years of age (N = 1083; 506 girls and 577 boys). Follow-up surveys were conducted when the participants were 18 (1983), 21 (1986), 30 (1995) and 43 (2008) years of age. In this report, data from the 1981, 1986, 1995 and 2008 surveys are presented. The cohort has in various comparisons been found to be representative of the Swedish population [26]. Of the original cohort, there were 1071 subjects still alive in 2008, of which 1003 (93.7%) agreed to participate in the survey at age 43. Due to non-response on one or more key measures (see below), the effective sample size of the present report is n = 824 (77%). All participants provided informed consent at all surveys and the study was approved by the Regional Ethical Review Boards in Umeå. For more details about the study, see Hammarström [26] and Hammarström & Janlert [27].

Participants completed a comprehensive questionnaire at all follow-ups. Although the composition of the questionnaire varied at different ages due to the age-specific relevance of some topics, the main areas covered in all versions included health and health behaviors, social and socioeconomic conditions, school/working conditions and leisure activities. The majority of items originated from the Swedish Survey of Living Conditions [28] and the Low-Income Study [29]. In 2008, blood samples were collected during a health examination.

## Measures

### Exposure: body size at birth

Delivery records of the participants' mothers from 1965 (i.e., pertaining to the participants' own birth) were retrieved from the archives of the respective delivery ward. From the delivery records, information on birth weight, length, date of delivery, date for last menstruation prior to delivery, and diagnoses according to the Swedish adaptation of the 6th revision of the International Classification of Disease [30], was documented.

Birth weight (BW) (kg), Birth length (cm), and ponderal index (weight/length^3^, kg/m3) were initially used as the main continuous measures of body size at birth. Since both ponderal index and birth length were less consistently related to lipid levels than was BW in preliminary analyses, these alternative measures were excluded from further analyses. Preterm or premature birth (defined as either a diagnosis of immaturity or birth < week 37, or if missing record data proxied as birth weight < 2500 g) did not influence the estimated associations, why all cases were included in the analyses. The associations between birth weight and lipids appeared to be approximately rectilinear by graphical inspection.

### Exposure: cumulative adversity over the life course

Adversity was defined as potentially stressful environmental exposures. Items representing this concept were selected from the questionnaires at each age, while items substantially dependent on subjective appraisal of the exposure were not selected. Since different versions of the questionnaire were used at different ages, the set of adversity components also varied between measurements. All non-binary components were dichotomized as close to the 80th percentile as possible. All components were then added generating an index of *cumulative adversity*. The following components of adversity were included (k = 28):

*Parental illness (age 16) *was defined as one or both parents having a physical illness, mental problems, and/or alcohol or drug problems, as reported by the adolescent.

*Parental loss (age 16) *was defined as either having experienced parental separation/divorce (n = 181), or parents never living together (n = 17), or death of either parent (n = 28).

*Parental unemployment (age 16) *was defined as one or both parents being unemployed or granted a disability pension at the time of the survey (housewives were classified as employed).

*Residential crowding (age 16) *was defined as the participant not having her/his own room.

*Residential mobility (age 16 and 21)*. The participants were asked how many times they had moved in their lifetime (at age 16) and during the last three years (at age 21). High residential mobility was defined as > 2 moves (= 1), compared to 0-2 moves (= 0) at each time period.

*Unemployment (age 21, 30 and 43) *was defined as current own unemployment or disability pension, based on a question about current labor market situation.

*Spousal unemployment (age 30 and 43)*. The participants were asked if their partner had been unemployed during the last five years (age 30), and about the partner's current labor market situation (age 43). In the latter case, unemployment or disability pension was considered unemployment.

*Low cash margin (age 21, 30 and 43)*. The participants were asked if it would be possible for them to raise a certain amount of cash within a week [31]. The amount was 5,000SEK at age 21, 13,000SEK at age 30, and 15,000SEK at age 43.

*Low income (age 21) *was defined as a self-reported monthly income <3,400SEK.

*Financial strain (age 30 and 43)*. The participants were asked how often they were forced, due to financial reasons, to abstain from any out of eleven different material needs (eat a cooked meal; buy clothes; pay the rent or other invoices; go to the movies, concert, theatre; invite relatives or friends; buy presents; go away on vacation; subscribe to a magazine; spare time activities or hobbies; go to a restaurant or pub). The response options were 'often', 'seldom', 'never' or 'not applicable'. The number of 'often' responses was added up to an index and dichotomized at the 80^th ^percentile, resulting in 3 or more frequent cut-backs considered as high financial strain at age 30, and one or more at age 43.

*Illness and death of close ones (age 21, 30 and 43)*. The participants were asked if someone close had had a serious or long-term illness, and if someone close had died, during the last three years (age 21), 12 months (age 30), or five years (age 43). A positive response was considered an adversity, separately for illness and death.

*Separation (age 30 and 43) *was defined as having broken up from a long-term relationship involving co-habituation during the last 12 months (at age 30) or since the age of 30 (at age 43).

*Bullying or sexual harassment at work (age 30 and 43)*. The participants were asked how often they had been exposed to personal persecution at the workplace through mean words and actions from bosses or colleagues, or exposed to sexual harassment through unwelcome or degrading sexual insinuations. Due to the low frequencies, a couple of days per months of either bullying or sexual harassment was considered exposure (= 1).

*Exposure to threat and violence (age 30 and 43)*. The participants were asked if they had been exposed to threats of violence that were so serious that she or he was scared, and if they had been exposed to physical violence, during the last 12 months. Due to low frequencies, a positive response on either violence or threat was considered exposure (= 1).

*Social isolation (age 30 and 43)*. The participants responded to four questions from the Availability of Social Integration (AVSI) scale [32,33], concerning the number of people available for emotional and social support. A total score of <80^th ^percentile was regarded as exposure to social isolation.

*Low decision latitude (age 30 and 43)*. Participants responded to 6 items about decision latitude from the Swedish Demand-Control Questionnaire (DCQ) [34], with responses on a four-level Likert scale, which were added together. The group belonging to the 20^th ^percentile was considered exposed to low decision latitude.

### Exposure: cumulative socioeconomic disadvantage over the life course

Participants' own occupation at age 21, 30 and 43 were coded according to the socioeconomic classification system of Statistics Sweden [35]. The classification is based on the main divisions of manual workers, non-manual employees and self-employed. Manual workers were categorized as low SES (= 1) while non-manual employees and self-employed were categorized as high SES (= 0). For participants who were not currently working and for whom information on previous occupation was not available (only at age 21 and 30), e.g. for participants who were unemployed, studying or doing military service, the highest educational attainment was used as a proxy (n = 206 for SES at age 21, n = 41 for SES at age 30): university-preparatory high school or university studies indicated high SES (= 0) while other types of high school education or lower were classified as low SES (= 1). At age 16, parental occupation was coded into three social groups. Having both parents in 'social group 3', comparable to manual workers, defined low SES (= 1), while having at least one parent in social group 1 or 2 defined high SES (= 0).

*Cumulative socioeconomic disadvantage *was operationalized as the number of life course periods with low SES (range 0-4).

### Current health behavior and body size at age 43

Self-reported health behavior at age 43 was examined as potential confounders: daily smoking (yes/no), daily snuff use (yes/no), alcohol consumption. Alcohol consumption (average gram pure alcohol consumption per week) was estimated from questions on the frequency of alcoholic beverage consumption, and approximate volume of beverage per occasion (separate items for beer, wine and spirits) and dichotomized at 110 g/week for men and 80 g/week for women [36]. At age 43, weight and height was measured according to the MONICA manual [37] as a part of a health examination at the participant's respective health care center. From this information BMI (kg/m^2^) was calculated. For those who had not completed weight and height measurement (n = 9), BMI calculations were based on self-reported height/weight. There was a high correspondence between measured and self-reported height, r(891) = .91.

### Outcome: blood lipids at age 43

At age 43, participants were invited to a health examination at their local health care center, including blood sampling after an overnight fast. Samples were assessed with respect to total cholesterol (TC), low-density lipoprotein cholesterol (LDL-C), high-density lipoprotein cholesterol (HDL-C), triglycerides (TG) Apolipoprotein (Apo) A1 and Apo B (for brevity, all outcomes including apolipoproteins are collectively referred to as 'lipids' in the text), according to the routine methods at the Department of Clinical Chemistry, Umeå University Hospital, Sweden. TG concentrations were logarithmically transformed by the natural logarithm prior to analysis to achieve a normal distribution. The other outcomes displayed approximately normal distributions.

*Dyslipidemia *was defined according to the 2003 European Society of Hypertension-European Society of Cardiology guidelines [38]: total cholesterol >6.5 mmol/L, or LDL cholesterol > 4.0 mmol/L, or HDL cholesterol <1.2 mmol/L for women and <1.0 mmol/L for men. This definition was used as a measure of high-risk dyslipidemia, rather than more recent guidelines which include individuals of lower risk [39] (in the present sample 71.5% were classified as having dyslipidemia according to the more recent criteria). Participants with medication against dyslipidemia (n = 28) were also classified as having dyslipidemia.

### Data analysis

There were 1003 participants in the study of which 888 had at least one valid lipid measure. Of these n = 840 had all six measures available as well as information on birth weight and life course exposures, comprising our effective sample. Due to missing data on either birth weight or questionnaires, the effective n went down to n = 824 (396 women, 428 men) when all variables were entered into the analysis (77% of the original cohort still alive). See Table 1 for descriptive statistics.

**Table 1:** Descriptive statistics of the main variables in women, men and in the total sample.

Variable	Women		Men		Total sample		
			
	Estimate	N	Estimate	N	Estimate	N	P value^1^
Exposures							

Birth weight (kg)	3.30 (0.53)	473	3.47 (0.60)	517	3.39 (0.57)	990	< .001

Cumulative low SES	1.64 (1.32)	461	1.91 (1.41)	503	1.78 (1.38)	964	.003

Cumulative adversity	6.52 (3.50)	482	5.65 (3.14)	521	6.07 (3.34)	1003	< .001

High alcohol, n(%)	38 (8.0)	477	64 (12.5)	510	102 (10.3)	987	.018

Smoking, n(%)	113 (23.7)	477	90 (17.7)	508	203 (20.6)	985	.021

Snuff use, n(%)	62 (13.0)	478	156 (31.1)	502	218 (22.2)	980	< .001

Outcomes							

Dyslipidemia, n(%)	143 (33.7)	424	212 (46.7)	454	355 (40.4)	878	< .001

Total cholesterol	5.21 (0.93)	424	5.50 (0.99)	462	5.36 (0.98)	886	< .001

Triglycerides^2^	1.07 (0.61)	423	1.71 (1.28)	455	1.40 (1.06)	878	< .001

HDL cholesterol	1.52 (0.37)	422	1.20 (0.32)	453	1.35 (0.38)	875	< .001

LDL cholesterol	3.19 (0.80)	420	3.54 (0.87)	433	3.36 (0.86)	853	< .001

Apo A1	1500 (253)	417	1365 (199)	445	1431 (236)	862	< .001

Apo B	914 (215)	417	1034 (231)	445	976 (231)	862	< .001

Estimates are mean (standard deviation) if not noted otherwise.

^1 ^Comparison between women and men; χ^2 ^test or t test.

^2 ^Descriptive statistics based on untransformed concentrations

Those with missing lipid data (n = 115) did not differ significantly from those with available lipid data (n = 888) regarding birth weight (t test; p = .09; M = 3.48 and 3.38 kg, respectively), cumulative adversity (p = .16) or cumulative SES (p = .60). Those with dyslipidemia medication (n = 28) did not differ from those without medication regarding SES, adversity or birth weight (all ps > .10), and where therefore included in the analyses.

Since different life course models might be relevant to explain socioeconomic influences on health, we employed a formal method recently proposed by Mishra et al. [40] for comparing the fit of different life course models. The method is based on a partial F test of a saturated model including all possible parameters (including interactions), and a reduced nested model where restrictions have been imposed on the saturated model to correspond to a specific life course model [40]. In our case we tested the cumulative risk and the early critical period models for the different lipid outcomes in sex-stratified analyses. In these analyses, all F-statistics were non-significant (F(14,380-412) < 1.52) for both the cumulative risk and the critical period model (data not shown), indicating that the restriction of either model did not confer any significant reduction in model fit. For consistency, we chose the more parsimonious cumulative risk formulation to represent life course SES throughout the analyses.

As our main analyses, associations between dyslipidemia and birth weight, cumulative socioeconomic disadvantage and adversity and current health behavior, were first examined by means of logistic regression analysis; results are presented as crude and adjusted Odds Ratios (OR) and 95% confidence intervals (CI). This general approach was then extended to specific examinations of the serum levels of each lipid. For these analyses, hierarchical linear regression was used, with continuous lipid measures at age 43 regressed on birth weight (Model 1), with the subsequent addition of cumulative socioeconomic disadvantage and adversity (Model 2), and current health behavior (Model 3). Additional contribution provided by each step was appraised by the ΔR^2 ^with corresponding F test. A higher ΔR^2 ^provided by the first step than in the second step would indicate that birth weight plays an important role for adult lipids while life course exposures are of less importance. The reverse situation would point to life course exposures being of larger importance. Standardized regression coefficients (β) are presented in addition to unstandardized regression coefficients (b) and standard errors (SE), to provide comparable estimates for the independent variables measured on different scales. A substantial attenuation of the birth weight coefficients with the adjustment for life course exposures could be an indication of the latter acting as confounders or mediators. Attenuation of life course coefficients in the third step would indicate that the association might be explained by behavioral pathways. Since adult BMI could act as a mediator or a confounder of birth weight [13] as well as of life course circumstances [41], in a complementary final model adjustment was done for BMI at age 43.

Analyzes were conducted on the whole sample as well as on women and men separately. SPSS version 17.0 was used for all analyses.

## Results

### Dyslipidemia, birth weight and life course exposures

As can be seen in Table 2, birth weight was not related to dyslipidemia at age 43. Cumulative socioeconomic disadvantage and adversity were both positively related to dyslipidemia in the total sample. These associations seemed to be driven by results in women, while in men neither variable were related to dyslipidemia. The individual ORs were attenuated below significance by mutual adjustment, and further attenuated by the addition of health behavior.

**Table 2:** Summary of logistic regression analyses with dyslipidemia on birth weight, cumulative socioeconomic disadvantage and cumulative adversity.

Predictor	Model 1	Model 2	Model 3
	**OR (95% CI)**	**OR (95% CI)**	**OR (95% CI)**

Total sample			

Birth weight	1.01 (0.80-1.28)	0.99 (0.77-1.27)	1.00 (0.78-1.28)

Low SES	1.12 (1.01-1.24)	1.08 (0.98-1.20)	1.06 (0.96-1.18)

Adversity	1.05 (1.00-1.09)	1.04 (0.99-1.08)	1.02 (0.98-1.07)

Women			

Birth weight	0.92 (0.63-1.34)	0.95 (0.63-1.43)	0.93 (0.62-1.41)

Low SES	1.24 (1.06-1.45)	1.15 (0.97-1.37)	1.13 (0.95-1.35)

Adversity	1.07 (1.01-1.14)	1.05 (0.99-1.12)	1.04 (0.98-1.11)

Men			

Birth weight	0.95 (0.70-1.30)	0.90 (0.65-1.25)	0.92 (0.66-1.27)

Low SES	1.01 (0.88-1.15)	0.99 (0.87-1.13)	0.97 (0.85-1.11)

Adversity	1.04 (0.98-1.11)	1.05 (0.98-1.12)	1.03 (0.96-1.10)

Model 1 = Bivariate (crude ORs) Model 2 = Birth weight, SES and adversity, Model 3 = Model 2 + health behavior (smoking, snuff use, high alcohol consumption; estimates not shown).

### Serum lipid levels, birth weight and life course exposures

As an initial examination of linear relationships between the exposures and the lipid levels, bivariate correlations between the continuous variables of interest are shown in Table 3. As can be seen in the table, birth weight was only weakly associated with most of the outcomes, with the exception for triglycerides in women which showed a negative association with birth weight. The life course exposures (cumulative low SES and adversity) mainly seemed to be of importance for women. In women, there were also weak correlations between birth weight and the life course exposures.

**Table 3:** Zero-order correlations between the main variables, in women (below diagonal) and men (above diagonal).

Variable	**1**.	**2**.	**3**.	**4**.	**5**.	**6**.	**7**.	**8**.	**9**.	**10**.	**11**.	**12**.
1. Birth weight	-	-.02	.03	.08^t^	.03	-.03	.08	-.03	.09^t^	.08^t^	.09^t^	.03

2. Low SES	-.09^t^	-	.18***	.07	.15***	.14**	-.01	.07	-.07	-.02	-.02	.04

3. Adversity	-.11*	.38***	-	.09^t^	.22***	.08^t^	.00	.08^t^	.02	-.01	.01	.05

4. Alcohol	.03	-.02	.02	-	.11*	.04	.09^t^	.13**	.04	.06	.06	.11*

5. Smoking	-.01	.24***	.26***	.09^t^	-	.21***	.13**	.18***	-.04	.07	-.01	.19***

6. Snuff	.01	.08	.07	.07	.02	-	.02	.11*	.02	-.02	.01	.02

7. Total cholesterol	-.08^t^	.01	.05	.01	.06	.05	-	.41***	-.03	.94***	.11*	.90***

8. Triglycerides	-.15**	.11*	.17***	.00	.15**	.02	.44***	-	-.38***	.23***	-.24***	.55***

9. HDL cholesterol	.04	-.14**	-.15**	.13**	-.12*	.06	.17***	-.31***	-	-.14*	-.88***	-.26**

10. LDL cholesterol	-.05	.05	.08^t^	-.04	.09^t^	.03	.91***	.34***	-.14**	-	-.06	.87***

11. Apo A1	-.02	-.07	-.11*	.13**	-.07	.09	.27***	-.06	.83***	-.05	-	-.12*

12. Apo B	-.09^t^	.08	.14**	-.04	.16**	.06	.85***	.58***	-.22***	.87***	-.01	-

These bivariate analyses were extended to hierarchical linear regression analyses. Total cholesterol was not explained by the set of predictors to any important degree, in either women (final model R2 = .012, p = .584) or men (final model R2 = .028, p = .062; current smoking being the only significant independent variable (p = .009)). Similarly, LDL-C was not related to any of the independent variables in women (final model R2 = .020, p = .246) or men (final model R2 = .017, p = .341). Summaries of the results for the remaining outcomes are found in table 4 (total sample), table 5 (women) and table 6 (men), overall displaying mixed findings.

**Table 4:** Summary of hierarchical regression models in the total sample: serum lipids on birth weight in model 1, adding life course exposures (cumulative socioeconomic disadvantage (SES) and cumulative adversity) in model 2, and current health behavior (smoking, snuff use, high alcohol consumption; estimates not shown) in model 3.

Criterion Predictor	Model 1 = birth weight				Model 2 = + life course exposures				Model 3 = + current health behavior			
					
	ΔR2 (p)	b (SE)	β(p)		ΔR2 (p)	b (SE)	β(p)		ΔR2 (p)	b (SE)	β(p))	Final R2 (p)
HDL-C	.000 (.907)				.018 (.001)				.004 (.318)			.023 (.005)

Birthweight		0.00 (0.02)	.00 (.907)			0.00 (0.02)	.00 (.977)			-0.00 (0.02)	-.00 (.963)	

SES						-0. 04 (0.01)	-.14 (< .001)			-0.04 (0.01)	-.13 (< .001)	

Adversity						0.00 (0.00)	.03 (.449)			0.00 (0.00)	.03 (.374)	

TG	.000 (.574)				.016 (.002)				.027 (< .001)			.043 (< .001)

Birthweight		-0.02 (0.04)	-.02 (.574)			-0.01 (0.04)	-.01 (.708)			-0.02 (0.04)	-.02 (.640)	

SES						0.05 (0.02)	.11 (.002)			0.04 (0.02)	.09 (.013)	

Adversity						0.01 (0.01)	.04 (.322)			0.00 (0.01)	.01 (.751)	

Apo A1	.000 (.988)				.005 (.154)				.004 (.324)			.009 (.301)

Birthweight		-0.22 (14.8)	.00 (.988)			-1.47 (14.8)	-.00 (.921)			-2.91 (14.8)	-.01 (.844)	

SES						-11.3 (6.18)	-.07 (.068)			-11.5 (6.25)	-.07 (.067)	

Adversity						-0.52 (2.61)	-.01 (.844)			-0.53 (2.67)	-.007 (.842)	

Apo B	.000 (.676)				.009 (.030)				.019 (.001)			.028 (.001)

Birthweight		6.01 (14.4)	.02 (.676)			8.19 (14.4)	.02 (.568)			7.91 (14.3)	.02 (.579)	

SES						13.2 (5.99)	.08 (.028)			9.76 (6.02)	.06 (.105)	

Adversity						2.40 (2.54)	.03 (.346)			0.40 (2.58)	.01 (.878)	

**Table 5:** Summary of hierarchical regression models in women: serum lipids on birth weight in model 1, adding life course exposures (cumulative socioeconomic disadvantage (SES) and cumulative adversity) in model 2, and current health behavior (smoking, snuff use, high alcohol consumption; estimates not shown) in model 3.

Criterion Predictor	Model 1 = birth weight				Model 2 = + life course exposures				Model 3 = + current health behavior			
					
	ΔR2 (p)	b (SE)	β(p)		ΔR2 (p)	b (SE)	β(p)		ΔR2 (p)	b (SE)	β(p))	Final R2 (p)
HDL-C	.003 (.296)				.029 (.006)				.032 (.005)			.061 (< .001)

Birthweight		0.04 (0.04)	.05 (.296)			0.02 (0.04)	.03 (.544)			0.02 (0.04)	.03 (.504)	

SES						-0.03 (0.02)	-.09 (.092)			-0.02 (0.02)	-.08 (.129)	

Adversity						-0.01 (0.01)	-.11 (.049)			-0.01 (0.01)	-.10 (.068)	

TG	.019 (.006)				.023 (.010)				.010 (.241)			.053 (.002)

Birthweight		-0.14 (0.05)	-.14 (.006)			-0.12 (0.05)	-.12 (.019)			-0.12 (0.05)	-.12 (.016)	

SES						0.02 (0.02)	.05 (.395)			0.01 (0.02)	.03 (.555)	

Adversity						0.02 (0.01)	.13 (.015)			0.02 (0.01)	.11 (.047)	

Apo A1	.000 (.883)				.012 (.100)				.033 (.005)			.045 (.007)

Birthweight		-3.62 (24.6)	-.01 (.883)			-10.9 (24.8)	-.02 (.660)			-10.4 (24.5)	-.02 (.671)	

SES						-5.44 (10.5)	-.03 (.604)			-4.58 (10.5)	-.02 (.662)	

Adversity						-7.04 (3.97)	-.010 (.077)			-7.20 (4.01)	-.10 (.074)	

Apo B	.006 (.122)				.020 (.020)				.022 (.033)			.048 (.004)

Birthweight		-32.3 (20.9)	-.08 (.122)			-24.3 (20.9)	-.06 (.246)			-26.5 (20.8)	-.06 (.203)	

SES						6.46 (8.83)	.04 (.465)			3.03 (8.86)	.02 (.733)	

Adversity						7.64 (3.35)	.12 (.023)			5.88(3.39)	.01 (.084)	

**Table 6:** Summary of hierarchical regression models in men: serum lipids on birth weight in model 1, adding life course exposures (cumulative socioeconomic disadvantage (SES) and cumulative adversity) in model 2, and current health behavior (smoking, snuff use, high alcohol consumption; estimates not shown) in model 3.

Criterion Predictor	Model 1 = birth weight				Model 2 = + life course exposures				Model 3 = + current health behavior			
					
	ΔR2 (p)	b (SE)	β(p)		ΔR2 (p)	b (SE)	β(p)		ΔR2 (p)	b (SE)	β(p))	Final R2 (p)
HDL-C	.008 (.070)				.007 (.249)				.007 (.419)			.021 (.180)

Birthweight		0.05 (0.03)	.09 (.070)			0.05 (0.03)	.09 (.077)			0.05 (0.03)	.09 (.083)	

SES						-0.02 (0.01)	-.08 (.109)			-0.02 (0.01)	-.08 (.104)	

Adversity						0.00 (0.01)	.034 (.487)			0.00 (0.01)	.041 (.411)	

TG	.001 (.583)				.012 (.074)				.040 (.001)			.053 (.001)

Birthweight		-0.03 (0.05)	-.03 (.583)			-0.03 (0.05)	-.03 (.582)			-0.03 (0.05)	-.03 (.556)	

SES						0.03 (0.02)	.08 (.117)			0.02 (0.02)	.05 (.279)	

Adversity						0.02 (0.01)	.07 (.160)			0.01 (0.01)	.03 (.528)	

Apo A1	.009 (.061)				.000 (.926)				.003 (.780)			.012 (.577)

Birthweight		30.7 (16.3)	.09 (.061)			30.6 (16.4)	.09 (.063)			30.3 (16.5)	.09 (.067)	

SES						-2.62 (6.80)	-.02 (.700)			-3.25 (6.89)	-.02 (638)	

Adversity						-0.05 (3.23)	0.00 (.989)			-0.12 (3.31)	-.00 (.971)	

Apo B	.001 (.625)				.002 (.612)				.040 (.001)			.043 (.004)

Birthweight		9.26 (18.9)	.02 (.625)			9.44 (19.0)	.03 (.619)			7.92 (18.7)	.02 (.673)	

SES						6.30 (7.88)	.04 (.424)			3.66 (7.83)	.02 (.640)	

Adversity						1.73 (3.75)	.02 (.645)			-1.13 (3.76)	-.02 (.763)	

In the total sample (table 4), birth weight was not related to any of the outcomes. Life course exposures, however, were independently related to HDL cholesterol, TG and Apo B (p change > .05). This was mainly explained by the contribution of cumulative SES, which for the most part also contributed independently of current health behavior (model 3).

In women (Table 5), birth weight was related only to triglycerides (p = .006), an association which remained largely unchanged throughout the subsequent steps, suggesting an independence from both life course exposures and health behaviors. Still, life course exposures (added in model 2) contributed slightly more than did birth weight (ΔR2 = .023 vs .019). SES did not appear to be as important as in the total sample; instead, the contribution of life course exposures was largely explained by cumulative adversity. To verify that the effect of birth weight was independent from socioeconomic disadvantage we re-run the second model with saturated SES formulation rather than the cumulative risk formulation (data not shown), resulting in no attenuation of the birth weight coefficient (b(SE) = - 0.013(0.05), p = .015). Similar contributions by life course exposures and adversity were apparent for HDL-C and Apo B, although slightly weaker and non-significant for Apo A1. These associations were somewhat attenuated by the addition of health behavior, indicating that the contribution by adversity was partly mediated by behavioral pathways.

In men (Table 6), associations were overall weaker and did not reach statistical significance, and life course exposures were non-significant. Birth weight displayed borderline significant relationships with HDL-C and Apo A1, independently of life course exposures and health behavior.

In complementary analyses, BMI at age 43 was added to the final model (Model 3) for each of the lipid outcomes (data not shown). BMI displayed a significant independent association with all lipids (all p < .001 except for total cholesterol: p = .006) in the total sample, and with all lipids (p < .001) except for total cholesterol (p > .10 in both women and men) and LDL-C in men (p > .10) in the sex-stratified analyses. The contribution of SES in the total sample was substantially attenuated by the addition of BMI, for HDL-C (β = -.07, p = .036), triglycerides (β = .03, p = .345) Apo A1 (β = -.02, p = .600) and Apo B (β = .02, p = .580). In women the significant/borderline significant independent contributions of adversity were unaffected by the addition of BMI for all lipids, as was the contribution of birth weight for triglycerides (β = .11, p = .018). In men, the near-significant contributions of birth weight were slightly accentuated and reached significance for both HDL-C (β = 10, p = .030) and Apo A1 (β = .10 p = .041). There were no other substantial changes of coefficients.

## Discussion

This study provides support for the hypothesis that the accumulation of unfavorable circumstances over the life course is related to adult dyslipidemia. Similar results were shown for serum levels of some lipids (HDL-C, triglycerides, Apo B) but not others (Total cholesterol, LDL-C, Apo A1). Associations were mainly found among women. Support for the fetal origins hypothesis was only found for triglycerides in women. Thus, life course exposures provided a more substantial overall contribution to adult serum lipids than did birth weight.

In the total sample, birth weight was not related to dyslipidemia or to any specific lipids, while cumulative socioeconomic disadvantage displayed significant associations with dyslipidemia, HDL-C, triglycerides and Apo B. For dyslipidemia, the independent contribution of SES and adversity were nonsignificant after mutual adjustment, possibly due their interrelationship, and the ORs were further attenuated by the addition of health behaviors. For HDL-C and triglycerides these associations seemed to be independent of current health behavior. These results support previous indications of weak and inconsistent influences of birth weight on lipids [13], and generally give support for the abundance of findings showing cumulative SES to be of importance for cardiovascular health [18].

The separate analyses on women and men provided a partly different picture. In women, triglycerides were significantly related to birth weight, an association independent of both social and behavioral factors. This is in line with previous findings indicating triglycerides to be the lipids with the most consistent association to birth weight [13], although similar results were not found in men. It has been suggested that since triglycerides are related to insulin, this lipid could indicate an aspect of extensive metabolic disorder including insulin resistance, and that this could be a reason as to why triglycerides are the lipids which show the most consistent association to birth weight [13]. It should be noted though, that even for triglycerides the contribution of life course exposures exceeded that of birth weight. This life course contribution was mainly explained by cumulative adversity, which also was the most important life course factor for the other lipid outcomes in women. However, although the independent contribution of SES was only near-significant, it should be noted that SES was related to HDL-C cholesterol and triglycerides in the bivariate analyses (Table 3). This association was attenuated when SES and adversity were simultaneously included in the model, probably as a consequence of the association between these two exposures (r = .38, Table 3). This pattern in women differed from that in men, where both birth weight and life course exposures displayed only weak associations with lipids and the strongest associations were found with health behaviors. Other studies have found social exposures over the life course to be of greater important for women than men [23,24,42,43]. This might be explained by structural patterns of unfavorable life circumstances which women are at greater risk for than men, in the present study supported by the substantially larger amount of adversities experienced by women than men (Table 1).

Adult BMI strongly attenuated the contribution of SES in the total sample, consistent with BMI acting as a mediator in the SES-lipids relationship. In men, the contribution of birth weight was slightly accentuated after the addition of BMI in adulthood, a phenomenon that has been highlighted in the literature on association between birth weight and lipids [13] and blood pressure [8]. Possibly, this could be explained by the contrasting effects of birth weight: birth weight is inversely associated with adult lipids, but positively associated with adult body mass, which in turn is positively related to lipids.

Total cholesterol and LDL cholesterol displayed no association with either birth weight or life course exposures. This is consistent with the observations that birth weight is only insubstantially related to total cholesterol levels [14]. Moreover, socioeconomic influences on total and LDL cholesterol are inconsistently reported in the literature [44].

### Methodological considerations

The main strengths of this study are the prospective design and high response rate. The high response rate, achieved despite the long-term prospective design, protects against selection bias which otherwise could influence the results [45]. In addition, missing data on the outcome measures, the chief source of internal drop-out, did not appear to be systematic with respect to any of the studied exposures. Assessment of birth weight based on birth records, as was done in this study, is preferable to self-reported retrospective recall, since the latter might be confounded by factors such as socioeconomic status or age at the time of reporting [46,47]. Also, a large part of the respondents might not be able to provide self-reports, which might introduce systematic error [46]. Error in self-report also tend to produce attenuation of observed associations [47]. Similarly, adult retrospective reports of adverse childhood experiences generally involve a large fraction of false negative responses and a considerable measurement error [48], which is why the prospective approach of the present study is preferable. These measurement issues are especially important in a study aiming at estimating the relative contribution of different exposures. Social deprivation has been shown to be related to birth weight [49], but there are also some reports of independent associations between birth weight and social circumstances later in life, e.g., unemployment in both women and men [50]. However, this association seems to be of less importance than social disadvantage in childhood [50].

The simple dichotomization of socioeconomic status at each age could possibly mask variation of SES important for the studied outcomes. However, this approach is a conventional operationalization of the cumulative risk model [51,52]. Although other conceptual life course models could have been examined, the cumulative risk model is the model that has received most support in the literature [18]. Our index of cumulative adversity differs from cumulative SES since it consists of exposures of widely different qualities, e.g. regarding the temporal demarcation, degree of threat, and social context of the adversities. This span of qualities should provide a reasonably comprehensive assessment of social adversities during the life course, although we do not have information on some salient exposures, e.g. particularly traumatic events in childhood, which might be of importance for adult health [53]. The simple number of adversities has been shown to be a practical way of summarizing total adversity exposure in relation to the health of children [54,55] and adults [21,56,57]. Nevertheless, our cumulative adversity measure is a crude operationalization and does not consider specific effects of individual adversities and timing of effects; such issues would be expected to confer a loss of power. Moreover, the lack of information on social circumstances during early childhood is a limitation of the study.

The meager findings concerning birth weight could be a result of too small a sample size and insufficient power. However, the aim of the study was to examine the relative contributions of different classes of exposures, and despite mainly non-significant results regarding birth weight, both life course exposures and health behaviors displayed more consistent associations with the outcomes. Thus, the relatively lower explanatory value of birth weight compared to life course exposures can still be interpreted. As noted, these findings are in line with previous research concluding that although there might be an influence of birth weight on adult cardiovascular risk factors, it is dubious that this potential effect is of substantial practical importance in this area [8,14].

## Conclusions

The accumulation of adverse social circumstances over the life course was found to be of relevance for dyslipidemia and serum levels of some lipids in adulthood, while the contributions of birth weight were inconsistent and where present, eclipsed by the stronger contribution of accumulated life course exposures. These results indicates that although there might be a weak influence of fetal conditions, social conditions and experiences over the life course do play a greater role for cardiovascular risk factors in adulthood.

## Competing interests

The authors declare that they have no competing interests.

## Authors' contributions

AH was responsible for the conception and design of the study and UJ and TT contributed to the design of the study. AH and UJ were responsible for the data collection. PEG was responsible for the statistical analyses and writing the first draft of the manuscript. All authors participated in interpreting the results, provided input on manuscript drafts, and read and approved the final manuscript.

## Pre-publication history

The pre-publication history for this paper can be accessed here:

http://www.biomedcentral.com/1471-2458/10/484/prepub
